# Near-Infrared Light-Responsive Shape Memory Polymer Fabricated from Reactive Melt Blending of Semicrystalline Maleated Polyolefin Elastomer and Polyaniline

**DOI:** 10.3390/polym13223984

**Published:** 2021-11-18

**Authors:** Min-Su Heo, Tae-Hoon Kim, Young-Wook Chang, Keon Soo Jang

**Affiliations:** 1 Department of Materials & Chemical Engineering, Hanyang University, Ansan 15588, Gyeonggi-do, Korea; su920122@hanyang.ac.kr (M.-S.H.); taehoonkim@hanyang.ac.kr (T.-H.K.); 2 BK21 FOUR ERICA-ACE Center, Hanyang University, Ansan 15588, Gyeonggi-do, Korea; 3 Department of Polymer Engineering, School of Chemical and Materials Engineering, The University of Suwon, Hwaseong 18323, Gyeonggi-do, Korea; ksjang@suwon.ac.kr

**Keywords:** shape memory polymer, NIR light responsive, semicrystalline maleated polyolefin elastomer, polyaniline, melt blending

## Abstract

A shape memory polymer was prepared by melt mixing a semicrystalline maleated polyolefin elastomer (mPOE) with a small amount of polyaniline (PANI) (up to 15 wt.%) in an internal mixer. Transmission electron microscopy (TEM), FTIR analysis, DMA, DSC, melt rheological analysis, and a tensile test were performed to characterize the structure and properties of the mPOE/PANI blends. The results revealed that the blends form a physically crosslinked network via the grafting of PANI onto the mPOE chains, and the PANI dispersed at the nanometer scale in the POE matrix served as a photo-thermal agent and provided increased crosslinking points. These structural features enabled the blends to exhibit a shape memory effect upon near-infrared (NIR) light irradiation. With increasing PANI content, the shape recovery rate of the blend under NIR stimulation was improved and reached 96% at 15 wt.% of PANI.

## 1. Introduction

A shape memory polymer (SMP) is a smart material that can memorize its original shape and recover to its original shape from temporarily fixed shapes, and responds to various types of external stimuli, such as heat, light, water, and electric or magnetic fields. Compared to metallic shape memory alloys, SMPs have several advantages, including low density, good processability, high deformability, biocompatibility, as well as easy control of transition temperature, which make these materials have various applications [1,2,3,4,5]. More extensive applications of SMPs were reported very recently, which include fabrications of smart surfaces with adjustable wetting properties [6], 3D structures with various complicated shapes [7], 4D-printed medical devices [8], and deployable solar arrays [9].

Among the various types of SMPs, light-responsive SMPs have gained interest due to several advantages they possess over conventional, thermally activated SMPs, such as remote activation ability and spatial controllability [10]. The most frequently employed approach to fabricate light-responsive SMPs is the incorporation of small amounts of photothermal reagents, such as gold [11], silver [12], titanium nitride [13], polydopamine [14], porphyrin [15], or carbon nanoparticles, such as CNT and graphene [16,17,18,19], into the shape memory polymer matrix. These additives act as photothermal heaters when the composites are irradiated with near infrared or sunlight, which enables the SMP nanocomposites to be indirectly heated above their transition temperature. For these composite systems, however, proper surface modifications of the fillers are required to enable them to be finely dispersed in matrix polymers. 

Conjugated polymers, such as polyaniline, polypyrrole, and polythiophene, show strong absorption of near-infrared energy, and transform it into thermal energy with high efficiency, which enables them to be used in photothermal therapy [20,21,22,23]. Only a few studies have been reported on light-responsive SMPs using these conjugated polymers as a photothermal agent [24,25,26]. Bai et al. reported an NIR-induced shape memory hydrogel by the incorporation of PANI nanofibers into polyvinyl alcohol (PVOH) by in situ polymerization of aniline in PVOH, where the PANI nanofiber served as a photothermal agent and provided increased crosslinking points [24]. Coating the PANI nanofiber onto a shape memory epoxy was also reported to exhibit NIR-responsive shape memory behavior [25]. Polypyrrole was incorporated into a shape memory polyurethane to prepare an NIR-induced shape memory elastomer [26]. Despite their excellent light-responsive behaviors, solution processing was needed to fabricate these SMPs, which was complicated and time consuming. It would be more practically useful if the photo-responsive SMPs based on conjugated polymers could be fabricated by a melt blending method.

PANI, which has a sulfonic acid group on its aromatic ring, is of particular interest, since it has a self-doped structure without the addition of any external dopants and exhibits strong absorption in the near-infrared (NIR) region [27,28,29], which makes it a promising photothermal agent. However, its use as a photothermal agent for the fabrication of light-responsive shape memory polymers has not been explored, especially by using a melt blending method. In this study, we observed that PANI obtained from oxidative chemical polymerization of aniline, possessing a sulfonic acid, can be covalently bonded onto a semicrystalline polyolefin elastomer, possessing a maleic anhydride group (mPOE), during melt processing, and is dispersed in the elastomer matrix at the nanometer scale. Further, the mPOE/PANI blends formed a physically crosslinked structure and exhibited photothermal behavior under NIR irradiation, which enabled these blends to have good light-responsive shape memory effects. Along with the examinations of photo-responsive shape memory effects, the thermo-mechanical and rheological properties of the blends were also investigated.

## 2. Materials and Methods

### 2.1. Materials and Sample Preparation

Semicrystalline maleated polyolefin elastomer (mPOE, maleic anhydride content 2 wt.%) was procured from DuPont (Fusabond N416, Wilmington, DE, USA). Aniline-3- sulfonic acid (ANISA, >99.0%) and ammonium persulfate (APS, 95%) were purchased from Sigma-Aldrich (St. Louis, MO, USA). Pyridine and acetone were procured from Junsei Chemical Co., Ltd. (Tokyo, Japan).

Polyaniline (PANI) was synthesized by oxidative chemical polymerization using ANISA as a monomer and APS as an oxidant as reported in the literature [27]. Briefly, 35.0 g (0.2 mol) of ANISA was dissolved in 100 mL of 3.0 mol/L pyridine aqueous solution at 4 °C. Further, 45.6 g (0.2 mol) of APS was dissolved in 180 mL of water. The APS solution was slowly added to the ANISA solution at a temperature below 4 °C for 1 h, and then the mixture was stirred for 15 h. The precipitate was collected and washed in acetone, and then dried under vacuum for 24 h at 30 °C.

mPOE/PANI blends with 3, 5, 10 and 15 wt.% PANI were prepared by melt blending in a Haake mixer (Haake Polylab Rheomix 600, Germany) for 10 min at 180 °C with a rotor speed of 60 rpm. The resulting mixtures were then molded as sheets by using an electrically heated hydraulic press at 180 °C for 10 min.

### 2.2. Characterization

Phase morphology of the blends was examined using transmission electron microscopy (TEM), with a JEOL 200CX TEM with an acceleration voltage of 200 kV. Ultra-thin sections were cut from the compression-molded specimens with a thickness of 100 nm using a Reichert ultracut cryo-microtome.

FTIR analysis was carried out using Bucker ALPHA spectrometer (Nicolet IS10, Thermo scientific, Waltham, MA, USA), equipped with an attenuated total reflectance (ATR) accessory, to characterize the PANI, and evaluate the possible interactions between the mPOE and PANI as well. The sample was placed on the ATR sample holder. The number of scans for each spectra was 16. The FTIR spectra were obtained in the range of 2000–700 cm^−1^ under nitrogen atmosphere. The empty sample chamber and ATR stage were used to obtain the background spectra.

Dynamic mechanical tests were carried out using a dynamic mechanical analyzer (TA Instrument, model DMA-Q800, Waltham, MA, USA). Samples were subjected to a cyclic tensile strain with an amplitude of 0.2% at a frequency of 1 Hz. The temperature was increased at a heating rate of 10 °C/min over the range from −100 to 150 °C.

Thermal properties of the samples were examined by using a differential scanning calorimeter (TA instruments, DSC Q20 equipped with RSC90 as a refrigerated cooling system). Samples were first heated from 30 to 200 °C at a rate of 10 °C/min under a nitrogen atmosphere and were kept for 5 min at this temperature to remove prior thermal history. The samples were then cooled down to −50 °C at a cooling rate of 10 °C/min (cooling scan), and were reheated to 200 °C at the same heating rate (second heating scan).

Tensile properties of the samples were measured by using a universal testing machine (UTM, AGS-500NX, Shimadzu, Japan) with a crosshead speed of 50 mm/min. The test was repeated at least five times at room temperature for each sample. 

Melt rheological measurements were conducted on an MCR 102 rheometer (Anton paar, Graz, Austria). Dynamic oscillatory shear measurements were performed at 180 °C using parallel plate geometry with a plate diameter of 25 mm and 1–2-mm-thick samples. The dynamic frequency sweep test was carried out between 0.1 and 100 rad/s. 

Photothermal effects of the blend were examined by measuring the surface temperature of the NIR-exposed sample using a digital thermometer during the NIR irradiation of the sample with 805 nm NIR at power density of 1.0 W/cm^2^ positioned at 30 cm from the sample. An infrared (IR) lamp with a red filter (Philips, Model Infraphil PAR38E, Berlin, Germany) was used as a light source. 

Shape memory behavior of the samples was evaluated using dog-bone-shaped specimens with a thickness of about 1 mm. Temporarily fixed sample was obtained by uniaxial deformation of the specimen to 50% at 70 °C (which is just above *T*_m_ of the sample) followed by cooling the deformed shape to 0 °C. Then, the temporarily deformed samples were exposed to NIR irradiation to analyze the shape recovery. The shape fixing ratio (*R*_f_) and shape recovery ratio (*R*_r_) of samples were determined by the following equations:

*R*_f_ (%) = [(*L*_s_ − *L*_i_)/(*L*_u_ − *L*_i_)] × 100 (1)

*R*_r_ (%) = [(*L*_s_ − *L*_r_)/(*L*_s_ − *L*_i_)] × 100 (2)

where *L*_i_ denotes the initial gauge length of the sample, *L*_u_ denotes the stretched length under load (1.5 × *L*_i_ in this experiment), *L*_s_ denotes the stretched length without load, and *L*_r_ denotes the recovered length upon NIR exposure, respectively. 

## 3. Results

### 3.1. Phase Morphology

**Figure 1:**
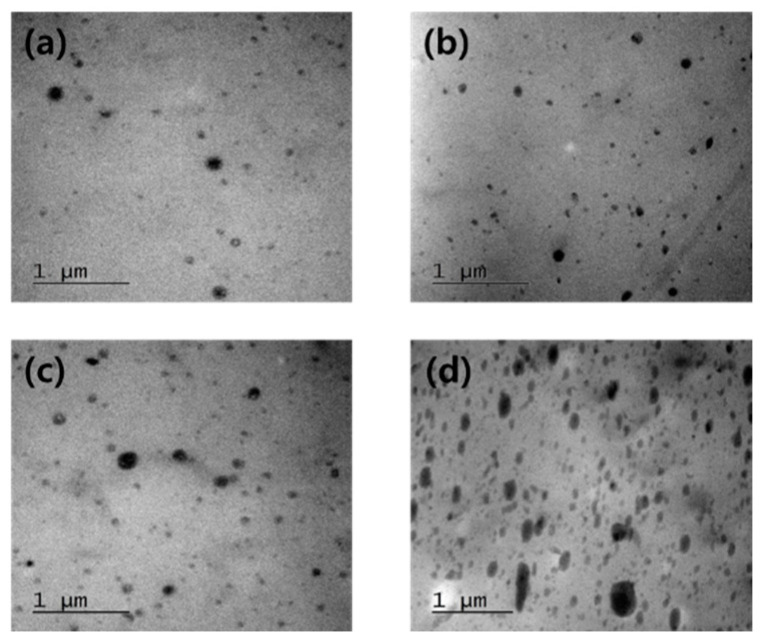
FE-TEM images of mPOE/PANI blends with (**a**) 3, (**b**) 5, (**c**) 10, and (**d**) 15 wt.% of PANI content (magnification ×50.0 k).

The phase morphology of the mPOE/PANI blends examined by TEM, and the TEM micrographs for the blends with various PANI contents are shown in Figure 1. A phase-separated morphology could be observed for all the samples in which the number of dispersed domains and their average diameter increased with increasing PANI contents in the blend, from about 100 nm for the blend with 3 wt.% of PANI to ca. 200 nm for the blend with 15 wt.% of PANI.

### 3.2. FT-IR

**Figure 2:**
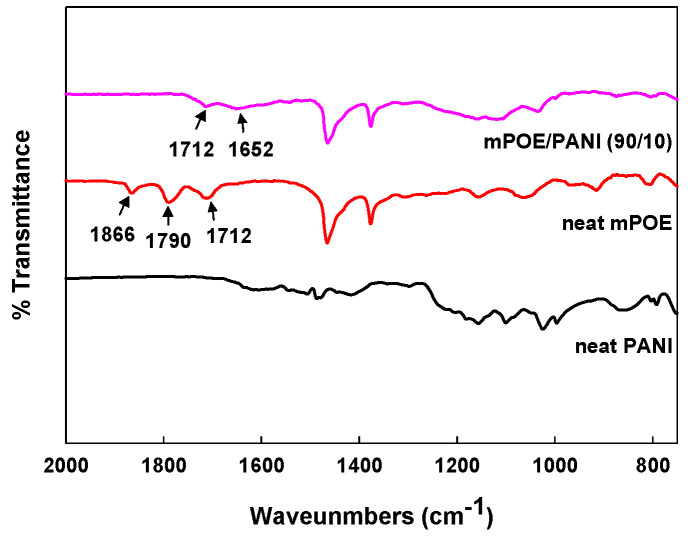
FT-IR spectra of neat PANI, neat mPOE and mPOE/PANI (90/10) blend.

FTIR characterization was performed to examine any possible interactions between mPOE and PANI in the blend. Figure 2 shows the FTIR spectra of neat mPOE and the mPOE/PANI (90/10) blend. The FTIR spectra of neat mPOE show characteristic absorption peaks at 1790 cm^−^^1^ and 1866 cm^−^^1^, attributed to the stretching vibration of cyclic maleic anhydride. The band at 1712 cm^−^^1^ was contributable to the stretching vibration of the carbonyl moiety attached to the cyclic maleic anhydride group [30]. In the FTIR spectra for the mPOE/PANI blend, a new absorption band, corresponding to 1652 cm^–^^1^, appeared, attributed to the C=O stretching of amide bonds, while there was a decrease in peak intensity, corresponding to maleic anhydride. This indicates that a chemical reaction occurred between the maleic anhydride of mPOE and the amine of PANI during the melt mixing process at high temperature. Such a reaction between PANI and the maleic anhydride group of other maleated polymers was also observed in blends of PANI with maleated PBT and maleated PS, in which PANI was grafted onto the polymer chains via the reaction [31,32]. It is thought that the nanoscaled phase-separated morphology achieved in the mPOE/PANI blends studied here was led by the reaction between the component polymers during melt blending. The nanostructured polymer blends obtained by reactive blending have been reported in other polymer blend systems, such as nylon/polyolefin blends [33,34] and PLA/PBAT blends [35]. 

### 3.3. Dynamic Mechanical Properties

**Figure 3:**
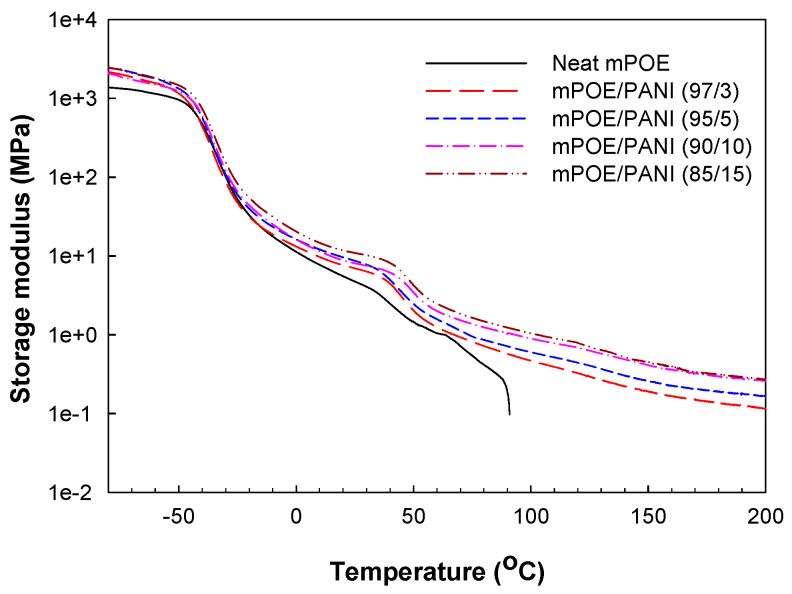
Variation in storage modulus (E′) with temperature for neat mPOE and mPOE/PANI blends.

Figure 3 shows the temperature dependency of storage modulus (E′) for neat mPOE and mPOE/PANI blends. The storage moduli for the blends are higher than those of the neat mPOE over the whole temperature range examined here, and the modulus increases with increasing PANI content in the blends, implying that the PANI nanoparticles acted as reinforcing fillers. It should also be noted that the storage modulus for neat mPOE decreases sharply when the temperature is higher than around 70 °C (which corresponds to *T*_m_), whereas the modulus of mPOE/PANI blends keeps quite stable up to 200 °C. Such a persistent modulus indicates that the blends form a crosslinked structure, which was obtained via the chemical reaction between the mPOE and PANI in the blends, as was confirmed by FTIR analysis. 

### 3.4. Thermal Properties

DSC analysis was performed to observe the variation in melting temperature (*T*_m_) and heat of fusion (Δ*H*_m_) with the composition of the blends, and those values and the associated degree of crystallinity (χ_c_) of the samples are listed in Table 1. The degree of crystallinity was calculated using Δ*H*_m_ per gram of mPOE obtained from DSC measurements and the ΔH_m_ corresponding to 100% crystalline low-density polyethylene (LDPE) (293 J/g) [30]. As illustrated in Table 1, the *T*_m_ and χ_c_ of the mPOE phase of the blends were continuously decreased upon increasing the PANI content in the blends. This suggests that the ordered crystalline structure of the semicrystalline mPOE was disturbed in the mPOE/PANI blends due to the formation of a crosslinked structure, which caused a decrease in the molecular mobility of the POE chains, and, thus, the crystallization is restricted, as manifested by the decrease in *T*_m_ and χ_c_ [36,37].

### 3.5. Tensile Properties

**Figure 4:**
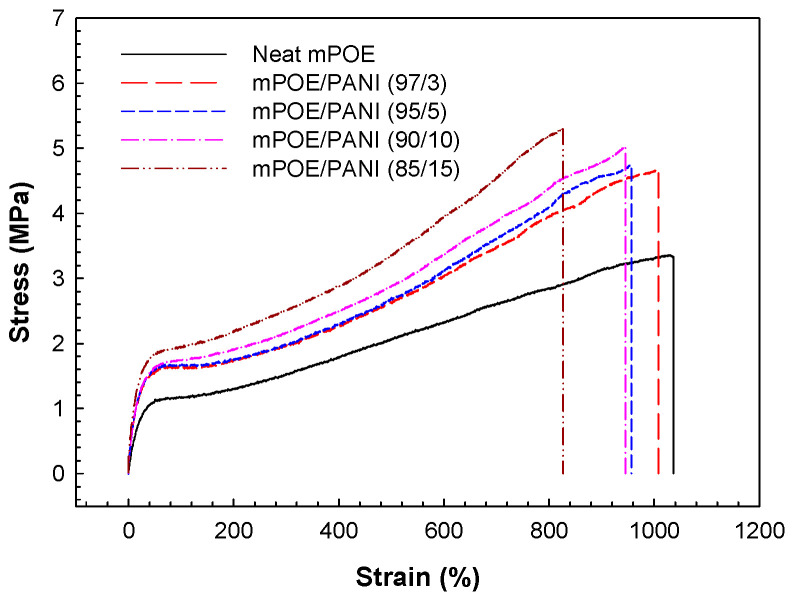
Stress–strain curves of mPOE/ PANI blends.

The stress–strain curves of neat mPOE and the mPOE/PANI blends are shown in Figure 4, and the results are summarized in Table 2. The tensile strength and modulus at a given strain of the blends are higher than those of neat mPOE, and the values increased with increasing PANI content in the blends. Neat mPOE exhibits typically elastomeric behavior, undergoing large deformation (about 1000% strain) with a low modulus. The blends exhibited similar deformation, along with a higher tensile modulus and strength, without serious loss of elongation at break, compared to neat mPOE. Such enhanced tensile properties are ascribed to the rigid nature of the PANI dispersed in the elastomer matrix, along with the strong interfacial adhesion between the two phases formed by covalent bonding, as discussed in the section above.

### 3.6. Melt Rheological Properties

**Figure 5:**
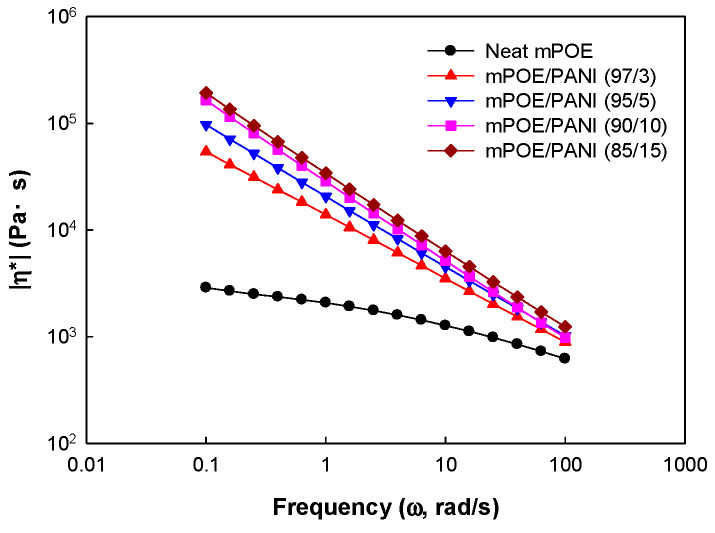
Variation in complex viscosity of mPOE/PANI blends with frequency.

It was observed that the blends are melt processable, even though they form a crosslinked structure, which suggests that the blends formed a physically crosslinked structure. In order to observe the effect of blend composition on melt rheological properties, melt viscosity was measured as a function of frequency by an oscillatory rheometer, and the results are presented in Figure 5. It can be observed that the melt viscosity of the mPOE/PANI blends showed a higher value compared to neat mPOE, especially at low frequencies, and it increased with increasing PANI content in the blends. Such enhanced viscosity of the mPOE/PANI blend indicated that the flow of POE chains in the molten state was restricted by the rigid PANI nanoparticles dispersed in the POE matrix. Additionally, all the blends showed typical shear-thinning behavior, in which viscosity decreases with increasing frequency, which is pronounced by increasing the PANI content in the blends. In general, the shear-thinning behavior is more pronounced in solid-like materials, such as the crosslinked polymer and polymer composites [38,39]. 

### 3.7. Photothermal Effects

**Figure 6:**
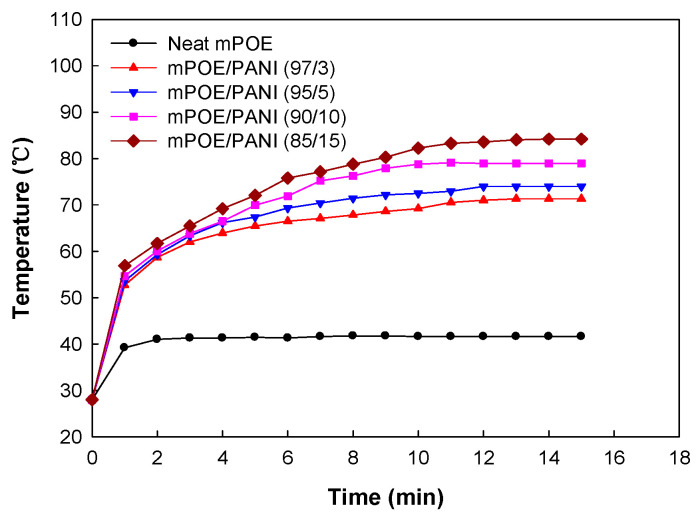
Variation in surface temperature with NIR irradiation time of mPOE/PANI blends.

A photothermal effect occurs when the exposed light is absorbed by a substance and is converted into thermal energy, thereby increasing the substance temperature. The photothermal effect of the samples was observed by measuring the surface temperature under irradiation of NIR light (1.0 W/cm^2^). Figure 6 shows the surface temperature of the pristine mPOE and mPOE/PANI blends as a function of NIR exposure time. The surface temperatures of the mPOE/PANI (85/15) blends increased from room temperature to 82 °C within 10 min of exposure, whereas the surface temperature of the neat mPOE only increased slightly. The results reveal that the PANI nano-domains dispersed in the POE matrix worked as photothermal nanoheaters, converting the imposed NIR to thermal energy.

### 3.8. NIR Light-Induced Shape Memory Effect

**Figure 7:**
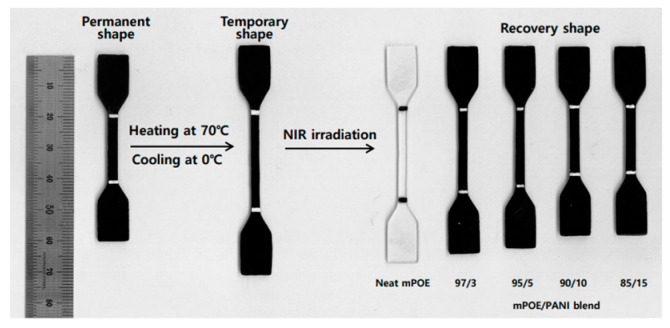
NIR light-induced shape recovery process of mPOE/ PANI blends.

As discussed above, the mPOE/PANI blends formed a crosslinked structure via the chemical reaction between PANI and semicrystalline maleated POE, and the modulus in the plateau region, which is related with crosslink density, increased with increasing PANI content. Further, the mPOE/PANI blends exhibited higher photothermal behavior with increasing PANI content. These results indicate that PANI acted as a crosslinking agent for the semicrystalline elastomer, as well as a photothermal nanoheater. These structural features of the blends enable them to exhibit shape memory behavior, i.e., crosslinked points stabilize the permanent shape, and a reversible phase, which has a crystalline melting transition, serves as a switch. A tensile recovery test was used to evaluate the light-induced shape memory effects of the samples. Shape recovery was observed to occur when the temporarily fixed specimen was exposed to NIR light, and the results are demonstrated in Figure 7, and the shape fixity (*R*_f_) and shape recovery (*R*_r_), calculated according to Equations (1) and (2), respectively, are shown in Table 3. It can be observed that *R*_r_ increased with increasing PANI content in the blends, and reached 96% for the blend with a PANI content of 15 wt.%. The improved shape recovery of the blends with a higher PANI content is ascribed to the increased crosslinked points, as well as the higher photothermal effects, as shown in the sections above. When the blend sample was deformed under a tensile load above its *T*_m_, elastic energy was stored during this deformation. Photothermal heating led to the melting of the crystalline domains of the temporarily fixed sample, and the polymer chains recovered to its original shape by releasing the stored energy. 

## 4. Conclusions

This study demonstrated that an NIR light-responsive shape memory elastomer can be fabricated by simple melt blending of a maleated semicrystalline polyolefin elastomer, with a small amount of polyaniline. Studies on phase morphology, thermo-mechanical analysis, and melt rheological analysis revealed that the blends formed a physically crosslinked semicrystalline polymer network, and the polyaniline nanodomains dispersed in the semicrystalline POE matrix acted as photothermal agents, as well as crosslinked points. Due to photothermal heating by the PANI nanoparticles, the crosslinked blends exhibited shape memory effects under NIR light irradiation. This novel shape memory elastomer may have potential applications, such as in remotely and spatially controllable soft actuators and grippers. 

## Figures and Tables

**Table 1:** Thermal characteristics of mPOE/PANI blends.

Sample	*T*_m_ (°C)	Δ*H*_m_ (J/g)	χ_c_ (%) ^1^
Neat mPOE	45.2	19.9	6.8
mPOE/PANI (97/3)	44.4	18.6	6.3
mPOE/PANI (95/5)	43.8	17.1	5.8
mPOE/PANI (90/10)	43.6	15.3	5.2
mPOE/PANI (85/15)	43.2	14.8	5.1

^1^ χ_c_ = 100 × (Δ*H*_m_/Δ*H*_m_**^◦^**)/w. Δ*H*_m_ and Δ*H*_m_**^◦^** are heats of melting for the sample and 100% crystalline LDPE (293 J/g), respectively. w: mass of the sample.

**Table 2:** Tensile properties of mPOE/PANI blends.

Sample	100% Tensile Modulus (MPa)	300% Tensile Modulus (MPa)	Tensile Strength (MPa)	Elongation-at-Break (%)
Neat mPOE	1.2 ± 0.1	1.5 ± 0.1	3.3 ± 0.2	1030 ± 70
mPOE/PANI (97/3)	1.6 ± 0.1	2.0 ± 0.2	4.6 ± 0.3	1000 ± 70
mPOE/PANI (95/5)	1.7 ± 0.1	2.0 ± 0.2	4.6 ± 0.3	950 ± 50
mPOE/PANI (90/10)	1.7 ± 0.1	2.2 ± 0.2	5.0 ± 0.3	940 ± 50
mPOE/PANI (85/15)	1.9 ± 0.1	2.5 ± 0.2	5.3 ± 0.3	820 ± 40

**Table 3:** Shape memory effects of mPOE/PANI blends.

Sample	*R*_f_ (%)	*R*_r_ (%)
Neat mPOE	98.2	28.5
mPOE/PANI (97/3)	99.6	44.6
mPOE/PANI (95/5)	99.7	88.5
mPOE/PANI (90/10)	99.7	92.9
mPOE/PANI (85/15)	99.6	96.2

## Data Availability

The data presented in this study are available on request from the corresponding author.
